# Attitudes towards statistics of graduate entry medical students: the role of prior learning experiences

**DOI:** 10.1186/1472-6920-14-70

**Published:** 2014-04-04

**Authors:** Ailish Hannigan, Avril C Hegarty, Deirdre McGrath

**Affiliations:** 1 Graduate Entry Medical School, University of Limerick, Limerick, Ireland; 2 Centre for Interventions in Infection, Immunity and Inflammation, University of Limerick, Limerick, Ireland; 3 MACSI, Department of Mathematics and Statistics, University of Limerick, Limerick, Ireland

**Keywords:** Attitudes, Statistics, Graduate entry, Medical education, Difficulty, Prior learning

## Abstract

**Background:**

While statistics is increasingly taught as part of the medical curriculum, it can be an unpopular subject and feedback from students indicates that some find it more difficult than other subjects. Understanding attitudes towards statistics on entry to graduate entry medical programmes is particularly important, given that many students may have been exposed to quantitative courses in their previous degree and hence bring preconceptions of their ability and interest to their medical education programme. The aim of this study therefore is to explore, for the first time, attitudes towards statistics of graduate entry medical students from a variety of backgrounds and focus on understanding the role of prior learning experiences.

**Methods:**

121 first year graduate entry medical students completed the Survey of Attitudes toward Statistics instrument together with information on demographics and prior learning experiences.

**Results:**

Students tended to appreciate the relevance of statistics in their professional life and be prepared to put effort into learning statistics. They had neutral to positive attitudes about their interest in statistics and their intellectual knowledge and skills when applied to it. Their feelings towards statistics were slightly less positive e.g. feelings of insecurity, stress, fear and frustration and they tended to view statistics as difficult. Even though 85% of students had taken a quantitative course in the past, only 24% of students described it as likely that they would take any course in statistics if the choice was theirs. How well students felt they had performed in mathematics in the past was a strong predictor of many of the components of attitudes.

**Conclusion:**

The teaching of statistics to medical students should start with addressing the association between students’ past experiences in mathematics and their attitudes towards statistics and encouraging students to recognise the difference between the two disciplines. Addressing these issues may reduce students’ anxiety and perception of difficulty at the start of their learning experience and encourage students to engage with statistics in their future careers.

## Background

Evidence based medicine has been defined as the ‘conscientious, explicit, and judicious use of current best evidence in making decisions about the care of individual patients’ [1]. Establishing best evidence involves systematically collecting and analyzing scientific evidence to answer a specific clinical question. Given the central role of statistics in the production and analysis of scientific data and in drawing inferences from that analysis, most medical students are expected to gain some knowledge of statistics as part of their education. A survey of practicing clinicians identified an understanding of probability and statistics as being useful for accessing clinical guidelines and evidence summaries, explaining levels of risk to patients, assessing medical marketing and advertising material, interpreting the results of a screening test, reading research publications for general professional interest, and using research publications to explore non-standard treatment and management options [2]. While statistics is increasingly taught as part of the medical curriculum, it can be an unpopular subject and feedback from students indicates that some find it more difficult than other subjects [3].

The teaching of statistics is not just about imparting knowledge but also about motivating students to continue to learn the quantitative skills they will need in their professional lives, so the role of attitudes towards statistics requires attention. Attitudes towards statistics are the extent to which learners hold positive or negative feelings towards statistics and their perception of its relevance, value and difficulty [4]. Positive attitudes towards statistics have been described [5] as the need for students to

• believe that they can understand and use statistics,

• think that statistics is useful in their professional lives,

• recognize that statistics can be interesting,

• be willing to invest the effort needed to learn statistical thinking and skills, and,

• realize that statistics is not easy but it also is not too difficult to learn.

The relationship between attitudes towards statistics and achievement in examinations is not clear with most studies only finding a small to moderate positive relationship between attitudes towards and performance in statistics [4,6]. Attitudes may, however, have a role in influencing the learning process and the willingness of students to engage with statistics outside the classroom and attend statistics courses in the future [7]. There has been considerable focus on attitudes towards statistics of secondary school and undergraduate students but little is known about attitudes towards statistics of postgraduates. Zhang et al. [8] explored the attitudes of postgraduate medical students in China and concluded that while the students had positive attitudes towards statistics, they perceived it as a difficult subject. Understanding attitudes towards statistics on entry to graduate entry medical programmes is particularly important, given that many students may have been exposed to quantitative courses in their previous degree and bring preconceptions of their ability and interest to their medical education programme. These preconceptions may be about mathematics rather than statistics which makes the teaching of statistics particularly challenging. Ben-Zvi and Garfield [9] refer to the tendency for students to ‘equate statistics with mathematics and expect the focus to be on numbers, computations, formulas, and on right answers’.

The aim of this study is to explore attitudes towards statistics of graduate entry medical students from a variety of backgrounds and focus on understanding the role of prior learning experiences. Understanding attitudes towards statistics on entry to the programme can inform the teaching of the discipline and addressing any issues at the start can help ensure a more positive experience for the students. The results of this study can also help inform those engaged in continuous professional development in medical research on the impact of previous learning experiences on attitudes towards statistics in the future.

## Methods

### Participants

The participants were enrolled in an exclusively graduate entry medical school in Ireland. All first year students were invited to participate in the study (n = 139). The instrument was distributed to the students in the first weeks of semester prior to any exposure to statistics in the programme to capture their attitudes towards statistics on entry to the programme. Participation was voluntary and anonymous. Ethical approval for this study was granted by the University Faculty Research Ethics committee.

### Instrument

Estrada et al. [10] list the 3 most widely used instruments to measure attitudes to statistics: Attitude toward statistics scale (ATS) [11]; Statistics Attitude Survey (SAS) [12]; Survey of Attitudes Toward Statistics (SATS) [13]. Because SATS has been used recently in a study of medical postgraduates [8], it was selected for use in this study to facilitate comparison across studies. The SATS-36 scale was used to measure six attitudes components i.e. Affect (students’ feelings concerning statistics); Cognitive Competence (students’ attitudes about their intellectual knowledge and skills when applied to statistics); Value (students’ attitudes about the usefulness, relevance, and worth of statistics in personal and professional life); Difficulty (students’ attitudes about the difficulty of statistics as a subject); Interest (students’ level of individual interest in statistics); and Effort (amount of work the student plans to expend to learn statistics). SATS-36 [14] is a recent extension of an earlier version of the SATS instrument which originally contained four of the components (Affect, Cognitive Competence, Value and Difficulty). A pre- and post-instruction version of the instrument is available. Given the timing of this survey before any formal statistics instruction in the graduate entry medicine programme, the pre- version of the instrument was used.

Responses to each of the 36 statements were on a scale from 1 (strongly disagree) to 7 (strongly agree). Of the 36 statements, 19 were negatively worded e.g. "I am scared by statistics". Statements which were considered to be negatively worded were reverse coded. A mean of the item responses for each component was obtained to give a measure on a scale of 1 to 7. Higher scores indicate more positive attitudes. Some additional information on demographics, primary degree and prior mathematical achievement were also asked in the survey instrument. Students were asked to rate their performance in mathematics in the past (school or college) on a scale of 1 to 7 where 1 represented very poorly and 7 represented very well. They were asked to give the number of quantitative modules (mathematics or statistics) in their primary degree. Students were also asked to rate their response to "If you had a choice how likely is it that you would have taken any course in statistics" on a scale of 1 to 7 where 1 is not at all likely and 7 is very likely.

### Statistical analysis

Cronbach’s alpha was used to measure the internal consistency of responses to all 36 items on the SATS instrument and items on the six components. Cronbach’s alpha was calculated using the reverse coded responses where appropriate. Multivariable linear regression was used to predict component scores using demographic variables i.e. age (< 25, ≥ 25), sex, nationality (Irish or non-Irish) and variables representing previous educational experiences: number of quantitative modules taken in their primary degree expressed as a binary variable representing none or one or more, perception of previous performance in mathematics (rated on a scale from 1 to 7). Assumptions underlying the model were tested. Spearman’s correlation coefficient was used to measure the strength of the association between rating of previous performance in mathematics, attitude components and the number of quantitative courses taken in their primary degree. All statistical analysis was carried out using IBM SPSS Statistics for Windows Version 20.

## Results

### Sample characteristics

Of the 139 students in first year, 121 responded to the survey giving a response rate of 87%. The majority (66%) of respondents were aged < 25; 69 (58%) were female; 70 (59%) were Irish and 37 (31%) were North American. The majority (61%) of respondents had a primary degree in science or engineering. 99 (85%) of the respondents reported having taken a quantitative course in their primary degree. The median number of quantitative courses taken was 2 (range 0–14).

### Internal reliability

Cronbach’s alpha was calculated using responses to all 36 items on SATS and indicated excellent reliability (alpha = 0.93). The reliability of the six components ranged from alpha = 0.79 for Value and Effort, 0.81 for Difficulty, 0.85 for Affect to 0.88 for Cognitive competence and Interest. All values indicated good reliability of the components and were similar to values of alpha reported in other studies [13,15].

### Scores on components by demographics and relationship with prior learning experiences

Table 1 gives the mean score for each component, perception of previous performance in mathematics and likelihood of choosing a course in statistics for all students, by demographic groups (age group, gender and nationality) and whether a student had taken a quantitative module in their primary degree. Students in our study tended to appreciate the usefulness and relevance of statistics in their personal and professional life (Value) and be prepared to put effort into learning statistics (Effort). They had neutral to positive attitudes about their interest in statistics (Interest) and their intellectual knowledge and skills when applied to it (Cognitive Competence). Their personal feelings towards statistics (as measured by the Affect component) were slightly less positive e.g. feelings of insecurity, stress, fear and frustration and they tended to view statistics as difficult (Difficulty).

**Table 1:** **Mean (SD) for SATS components and perceptions by demographics**^**1**^

	**Total (n = 121)**	**Males (n = 51)**	**Females (n = 69)**	**Irish (n = 70)**	**Other nationality (n = 49)**	**Age < 25 years (n = 79)**	**Age ≥ 25 years (n = 41)**	**No previous courses**^2^* (n = 17)	**Previous courses (n = 99)**
**Affect**	3.7 (1.26)	4.0 (1.24)	3.4 (1.22)	3.4 (1.27)	4.0 (1.15)	3.8 (1.18)	3.4 (1.37)	3.3 (2.40)	3.8 (1.21)
**Cognitive competence**	4.6 (1.25)	4.9 (1.17)	4.4 (1.28)	4.3 (1.33)	5.0 (0.99)	4.8 (1.16)	4.3 (1.37)	4.5 (2.30)	4.8 (1.17)
**Value**	5.1 (0.90)	5.2 (0.92)	5.1 (0.89)	5.0 (0.92)	5.3 (0.84)	5.2 (0.86)	5.1 (0.98)	5.0 (1.9)	5.2 (0.88)
**Difficulty**	3.4 (0.93)	3.5 (0.97)	3.3 (0.89)	3.2 (0.97)	3.7 (0.80)	3.6 (0.88)	3.0 (0.90)	2.9 (1.6)	3.5 (0.89)
**Interest**	4.7 (1.27)	4.8 (1.24)	4.6 (1.28)	4.5 (1.35)	4.9 (1.12)	4.6 (1.25)	4.8 (1.30)	4.2 (2.3)	4.7 (1.20)
**Effort**	6.0 (0.93)	6.0 (0.73)	6.1 (1.06)	6.0 (0.98)	6.1 (0.87)	6.1 (0.92)	5.8 (0.94)	6.2 (1.2)	6.1 (0.94)
Perception of performance in mathematics*	5.0 (2.0)	5.0 (2.0)	5.0 (2.0)	5.0 (2.0)	5.0 (2.0)	5.0 (2.0)	5.0 (2.0)	4.0 (3.0)	5.0 (2.0)
How likely to take a course in statistics*	2.0 (3.0)	3.0 (4.0)	2.0 (3.0)	2.0 (3.0)	3.0 (4.0)	2.0 (3.0)	2.0 (3.0)	2.0 (3.0)	3.0 (3.0)

^1^Some missing demographic data.

^2^*Results represent median (IQR).

The median response to the question "If you had a choice how likely is it that you would have taken any course in statistics" on a scale of 1 to 7 where 1 is not at all likely and 7 is very likely was 2 (Table 1), with 40 students (33%) responding not all at likely (rating = 1). Even though 85% of students had taken a quantitative course in the past, only 24% of students were positive towards taking one (score of 5 or above on a scale from 1 to 7).

The number of previous quantitative courses taken was weakly to moderately positively correlated with Difficulty and Cognitive Competence (Table 2) indicating that the more courses a student had taken, the less the perception of difficulty and the more positive the attitudes about their intellectual knowledge and skills when applied to statistics. A stronger relationship was found between perception of performance in mathematics in the past and all of the components of attitude apart from Effort (Table 2) indicating that the better the perception of previous performance in mathematics, the more positive the attitudes towards statistics. There was more variability in the component scores of students who hadn’t taken a quantitative module in their primary degree compared to all other subgroups (Table 1). This group also had the lowest median value for perception of previous performance in mathematics.

**Table 2:** Correlation of ranking of previous performance in mathematics, number of quantitative courses taken in primary degree and attitude components

**Attitude component**	**Previous performance in mathematics**		**Number of quantitative courses** **taken**	
	**r**_**s**_	**p-value**	**r**_**s**_	**p-value**
**Affect**	0.48	<0.001	0.16	0.10
**Cognitive Competence**	0.54	<0.001	0.31	0.001
**Value**	0.30	0.001	0.11	0.23
**Difficulty**	0.38	<0.001	0.27	0.004
**Interest**	0.25	0.007	0.05	0.63
**Effort**	0.09	0.35	0.08	0.42

Female students tended to score lower on all components apart from Effort compared to the male students but there were no significant gender differences for any of the six components, after adjustment for age group, nationality, whether or not a quantitative course had been taken in their primary degree and rating of performance in mathematics (Table 3). Irish students tended to score lower than students from other nationalities (mostly North American) on all components. In a multivariable regression model, being Irish or not was a significant predictor of Affect, Cognitive Competence, Value and Difficulty (Table 3). Older students also tended to score lower than younger students on all components apart from Interest. Being older was a significant predictor of Difficulty after adjustment for gender, nationality, whether or not a quantitative course had been taken in their primary degree and rating of performance in mathematics (Table 3). In a multivariable regression model, having taken a quantitative module in their primary degree was not a significant predictor of any of the attitude components. Perception of performance in mathematics in the past was, however, a strong predictor of all of the attitude components apart from Effort after adjusting for age group, gender, nationality and whether or not a quantitative course had been taken in their primary degree. Model fit was poor for some of the attitude components e.g. Value, Interest and Effort.

**Table 3:** Multiple linear regression models predicting attitude components

	**Outcome variable**											
	**Affect**		**Cognitive competence**		**Value**		**Difficulty**		**Interest**		**Effort**	
**Explanatory variable**	**β (SE)**	**p-value**	**β (SE)**	**p-value**	**β (SE)**	**p-value**	**β (SE)**	**p-value**	**β (SE)**	**p-value**	**β (SE)**	**p-value**
Female sex (reference male)	-0.38 (0.22)	0.09	-0.24 (0.19)	0.20	0.01 (0.18)	0.95	-0.05 (0.16)	0.75	-0.19 (0.24)	0.44	0.03 (0.18)	0.88
Irish nationality (reference non-Irish students)	-0.48 (0.22)	0.04	-0.59 (0.19)	0.003	-0.36 (0.18)	0.048	-0.47 (0.16)	0.004	-0.23 (0.24)	0.36	-0.19 (0.19)	0.30
Older age group (reference students aged < 25)	-0.07 (0.23)	0.76	-0.06 (0.20)	0.75	-0.03 (0.19)	0.87	-0.47 (0.17)	0.006	0.27 (0.26)	0.29	-0.29 (0.20)	0.14
Previous modules taken (reference none)	0.09 (0.31)	0.77	0.39 (0.27)	0.16	0.04 (0.27)	0.89	0.05 (0.23)	0.82	0.06 (0.35)	0.86	-0.07 (0.27)	0.79
Perception of performance in mathematics in the past	0.36 (0.07)	<0.001	0.44 (0.06)	<0.001	0.12 (0.06)	0.05	0.21 (0.05)	<0.001	0.24 (0.08)	0.005	-0.04 (0.06)	0.56
**Measure of goodness of fit (R**^**2**^**)**	0.25		0.40		0.04		0.26		0.06		0.03	

## Discussion

While the students in this study tended to perceive statistics as difficult, the perception of difficulty was not as strong as that reported by Zhang et al. [8] for a sample of Chinese medical postgraduates (a mean of 3.4 in this study compared to 2.9), however feelings towards statistics were less positive in our study compared to Zhang et al.’s (mean of 3.7 compared to 4.5). The majority of students in our study had a primary degree in science or a science related subject and had been exposed to quantitative modules (mathematics or statistics) in their primary degree. If the choice to do a course in statistics was theirs, however, only 24% of them described it as likely that they would take the course with 33% stating that it was not at all likely that they would take the course. There may be many reasons for this including a perception that they had already covered the material but this finding has implications for programmes which offer statistics as an option or elective and also for continuous professional development in medical research of which statistics is a critical component.

Zhang et al.’s study only included Chinese students but our study involves students from many different nationalities (mostly Irish or North American). Cultural differences were evident with Irish students less confident about their ability, placing less value on statistics and perceiving statistics as more difficult. While gender differences in attitudes towards statistics have been reported elsewhere [16], and female students in our study tended to score lower than the male students, no significant gender differences were found after adjusting for age, nationality, rating of previous performance in mathematics and whether a quantitative module had been taken in their primary degree. The generally less positive attitudes for older students and particularly their stronger perception of difficulty also have implications for engagement with continuous professional development during their careers.

The strongest predictor of most of the attitude components was how well students felt they had performed in mathematics in the past. This association of past experiences in mathematics with attitudes towards statistics needs to be addressed by statistics educators. Increasingly, statistics is viewed as a separate discipline rather than a subfield of mathematics [17]. Cobb and Moore [18] propose that ‘statistics requires a different kind of thinking, because data are not just numbers, they are numbers with a context’. Statistics did not originate in mathematics and the role of context, variability and data production in statistics differentiates statistical thinking from mathematical thinking. Statistical thinking also relies heavily on interpretation and critical judgement. There is increasing evidence that a strong background in mathematics does not necessarily translate to performance in statistics. Evans [4] demonstrated that students studying statistics in other disciplines such as sociology had more positive attitudes and correct conceptions of statistical fundamentals than mathematics students. Hannigan et al. [15] demonstrated that despite being very mathematically able and confident, a sample of prospective mathematics teachers did no better in an internationally used assessment of conceptual understanding of statistics than students from mostly non-quantitative disciplines. Highlighting the differences between mathematical thinking and statistical thinking may encourage medical students with negative experiences in mathematics in the past and poor perceptions of their ability in mathematics to ‘start afresh’ with statistics and be open to the idea that they will be able to use and understand statistics. Increasingly, statistics educators are encouraged to ground the teaching of statistics in context. Miles et al. [19] suggest that grounding the teaching of statistics in the context of medical research and typical clinical scenarios may better prepare medical students for their subsequent careers. Freeman et al. [3] demonstrated that a multidisciplinary approach to the teaching of medical statistics bringing together a statistician, clinician and educational experts to re-conceptualize the syllabus and placing greater emphasis on applying statistics and interpreting data can bring about better outcomes for students.

## Conclusions

The teaching of statistics to medical students should start with addressing the association between students’ past experiences in mathematics and attitudes towards statistics and encouraging students to recognise the difference between the two disciplines. Statistical thinking, which is different from mathematical thinking and focuses on context and variability, should be emphasized and the teaching of medical statistics should be grounded in context. Addressing these issues may reduce students’ anxiety and perception of difficulty at the start of their learning experience and encourage students to engage with statistics in their future careers.

## Abbreviations

SATS: Survey of Attitudes Toward Statistics; ATS: Attitudes Toward Statistics; SAS: Statistics Attitude Survey; SPSS: Statistical Package for the Social Sciences.

## Competing interests

The authors declare that they have no competing interests.

## Authors’ contributions

AH designed and conducted the study. AH and ACH conducted the statistical analysis. AH, ACH and DMCG contributed to the writing of the paper. All authors read and approved the final manuscript.

## Pre-publication history

The pre-publication history for this paper can be accessed here:

http://www.biomedcentral.com/1472-6920/14/70/prepub
